# A molecular and neuronal basis for amino acid sensing in the *Drosophila* larva

**DOI:** 10.1038/srep34871

**Published:** 2016-12-16

**Authors:** Vincent Croset, Michael Schleyer, J. Roman Arguello, Bertram Gerber, Richard Benton

**Affiliations:** 1 Center for Integrative Genomics, Faculty of Biology and Medicine, University of Lausanne, 1015 Lausanne, Switzerland; 2 Leibniz Institute for Neurobiology (LIN), Department Genetics of Learning and Memory, Brenneckestrasse 6, 39118 Magdeburg, Germany; 3 Otto von Guericke University Magdeburg, Institute for Biology, Behavior Genetics, Universitätsplatz 2, 39106 Magdeburg, Germany; 4 Center of Behavioural Brain Science (CBBS), Universitätsplatz 2, 39106 Magdeburg, Germany

## Abstract

Amino acids are important nutrients for animals, reflected in conserved internal pathways in vertebrates and invertebrates for monitoring cellular levels of these compounds. In mammals, sensory cells and metabotropic glutamate receptor-related taste receptors that detect environmental sources of amino acids in food are also well-characterised. By contrast, it is unclear how insects perceive this class of molecules through peripheral chemosensory mechanisms. Here we investigate amino acid sensing in *Drosophila melanogaster* larvae, which feed ravenously to support their rapid growth. We show that larvae display diverse behaviours (attraction, aversion, neutral) towards different amino acids, which depend upon stimulus concentration. Some of these behaviours require IR76b, a member of the variant ionotropic glutamate receptor repertoire of invertebrate chemoreceptors. IR76b is broadly expressed in larval taste neurons, suggesting a role as a co-receptor. We identify a subpopulation of these neurons that displays physiological activation by some, but not all, amino acids, and which mediate suppression of feeding by high concentrations of at least a subset of these compounds. Our data reveal the first elements of a sophisticated neuronal and molecular substrate by which these animals detect and behave towards external sources of amino acids.

Amino acids are vital for all organisms, both as constituents of proteins and as signalling molecules^1^. In animals, many of the twenty canonical L-amino acids that serve as building blocks for protein synthesis can be produced endogenously, but a subset must be obtained through their diet. Of these “essential” amino acids, nine are common to mammals and insects (histidine, isoleucine, leucine, lysine, methionine, phenylalanine, threonine, tryptophan and valine); insects also require a nutritional source of arginine^2^. In addition to a minimum basal intake, the precise ratio of dietary amino acids is crucial^3^,^4^,^5^.

Consistent with the fundamental importance of amino acids, the cellular mechanisms that sense these molecules are widely conserved. A central component of this pathway is the Target of Rapamycin (TOR) kinase, which integrates information from the levels of amino acids (and other environmental signals) to control cell growth and metabolism^6^. Signalling elements upstream of TOR that mediate uptake and/or direct detection of amino acids are, however, only starting to be identified; these include both substrate-specific cytosolic amino acid-binding proteins and transmembrane transporters^6^,^7^.

Because of the dietary requirement for many amino acids, animals have also evolved peripheral chemosensory pathways that can detect environmental sources of these nutrients and induce adaptive behaviours^8^. In humans, some, but not all, amino acids elicit an appetitive, savoury “umami” taste^9^, and rodents display diverse behaviours towards different amino acids^10^. The best-characterised amino acid sensory receptor in mammals is a heteromeric complex of the T1R1 and T1R3 G protein-coupled receptors, which are distantly-related to metabotropic glutamate receptors^11^,^12^. T1R1/T1R3 proteins are expressed in specific cells in lingual taste buds, and are required for amino acid-evoked electrophysiological responses in the lingual nerve^11^,^12^. Although this heteromeric receptor is a key mechanism for environmental amino acid sensing, the observed heterogeneity in perception suggests that these stimuli act through additional pathways.

Dietary amino acids are also critical for insects, notably to support a high rate of egg production in females^13^. Although there has been some investigation of behavioural responses of insects towards individual amino acids^14^, the sensory mechanisms allowing them to perceive these chemicals in the environment are largely unknown. Electrophysiological responses of chemosensory neurons in adult feeding organs to individual (or mixes of) amino acids have been described in several species^15^,^16^,^17^,^18^, but the behavioural roles of these neurons are unexplored. In the genetic model *Drosophila melanogaster*, where substantial progress has been made in revealing the molecular and neuronal basis of sweet and bitter tastants^19^, amino acid sensing mechanisms are surprisingly poorly understood. This might reflect, in part, the observation that adult *Drosophila* display robust behavioural responses towards amino acids only when previously deprived of these nutrients^20^, similar to locusts^18^. It remains unclear whether such plasticity in behaviour reflects the function of specific peripheral chemoreceptive pathways or internal metabolic amino acid sensors.

Larval *Drosophila* represent an appealing system for investigating sensory detection of amino acids, because these animals display a remarkably persistent appetite to support a 250-fold increase in mass from hatching to pupation. In this study, we have investigated the behavioural responses of larvae to amino acids and delineated some of the relevant chemosensory receptors and neurons.

## Results

### *Drosophila* larvae display innate, stimulus-specific responses to amino acids

**Figure 1:**
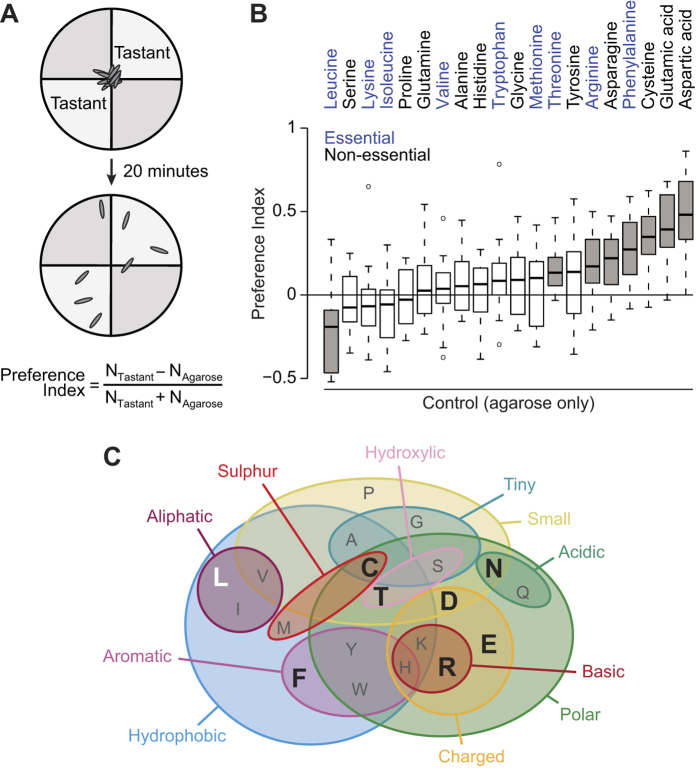
*Drosophila* larvae display innate, stimulus-specific responses to amino acids. (**A**) Schematic of the larval gustatory choice assay. Groups of ~30 experimentally-naïve larvae are placed at the centre of a Petri dish divided in four quadrants, which contain control (agarose alone) or test (agarose + tastant) substrates. The Preference Index is calculated by counting the number of larvae on each substrate and dividing the difference by the total number of larvae; positive values reflect behavioural attraction towards a tastant-containing substrate. **(B)** Preference Indices of *wild-type* larvae (*w*^*1118*^) for substrates containing individual L-amino acids (all at 50 mM, except for tyrosine, which was used at 2 mM because of its lower solubility). Essential amino acids are shown in blue. Grey boxes indicate amino acids that produce a Preference Index significantly different from 0 (t-test with Bonferroni correction, p < 0.0025). For boxplots in this and subsequent figures, the boxes represent the upper and lower quartiles, the thick line is the median value and the whiskers show the 5^th^ and 95^th^ percentiles. Circles outside the whiskers are outliers. (N ≥ 18 groups of ~30 larvae for each tastant). **(C)** Venn diagram (adapted from ref. 57) summarising the main physico-chemical properties of amino acids, indicated by single letter codes. Large black letters indicate those that are attractive to larvae; the sole aversive amino acid is shown in white.

We first asked whether larvae show innate behavioural responses to amino acids using a modified version of a paradigm for measuring taste preferences^21^. In brief, experimentally-naïve animals were allowed to wander freely on the surface of a soft agarose substrate in a four-quadrant circular arena, in which two opposing quadrants also contained a specific amino acid (Fig. 1A). The distribution of animals on quadrants containing or lacking the amino acid was quantified after 20 minutes and used to generate a Preference Index for the stimulus (Fig. 1A). Larvae display diverse behavioural responses to the 20 canonical L-amino acids (Fig. 1B). Seven amino acids promoted varying degrees of attraction (threonine, arginine, asparagine, phenylalanine, cysteine, glutamic acid and aspartic acid), one (leucine) was aversive, and the remainder were neutral. Essential amino acids are not confined to any particular category of response (Fig. 1B, blue labelling). Furthermore, we did not observe any clear relationship between physico-chemical properties of different amino acids and the behavioural responses they evoke; although 6 out of 7 appetitive amino acids are polar, six other polar amino acids are neutral (Fig. 1C). These results suggest the existence of multiple mechanisms that mediate different behavioural responses to distinct amino acid stimuli.

### IR76b is required for behavioural attraction to amino acids

**Figure 2:**
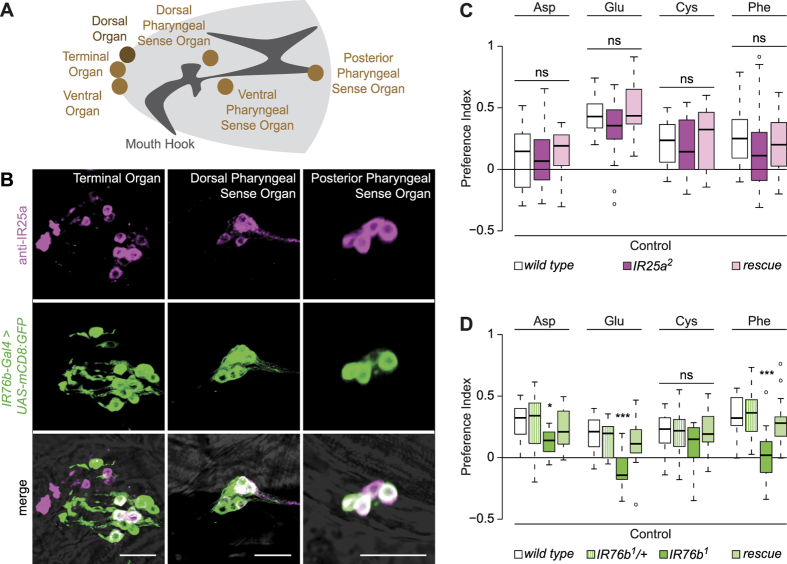
IR76b, but not IR25a, is required for behavioural attraction to amino acids. **(A)** Schematic of a larval head, illustrating the main olfactory (Dorsal Organ) and gustatory (Terminal Organ, Ventral Organ, Dorsal Pharyngeal Sense Organ, Ventral Pharyngeal Sense Organ, Posterior Pharyngeal Sense Organ) structures. **(B)** Immunofluorescence with anti-IR25a (magenta) and anti-GFP (green) on *IR76b-Gal4;UAS-mCD8:GFP* animals revealing expression in the indicated gustatory organs. The merged channels are overlaid on a brightfield image. Scale bars: 20 μm. **(C)** Preference Indices of *wild type (w*^*1118*^), *IR25a* mutants (*IR25a*^*2*^) and *IR25a* rescue animals (*IR25a*^*2*^*, CH322-32C20*) for the indicated amino acids (Kruskal-Wallis, p > 0.05; N ≥ 20 groups of ~30 larvae per genotype and per tastant). **(D)** Preference Indices of *wild type (w*^*1118*^), *IR76b* heterozygous (*IR76b*^*1*^*/*+; where “+” represents Canton S-derived chromosomes) and homozygous (*IR76b*^*1*^) mutants, and *IR76b* rescue animals (*IR76b-Gal4, UAS-IR76b;IR76b*^*1*^) for the indicated amino acids (Kruskal-Wallis, *p < 0.05, ***p < 0.001; N = 14 groups of ~30 larvae per genotype and per tastant).

Although central neuronal sensors for amino acids levels in the haemolymph have been identified in *Drosophila*^3^, it is unknown whether this class of molecules is also detected by peripheral chemosensory mechanisms. The larva possesses a variety of chemosensory organs located either externally (Terminal, Dorsal and Ventral Organs) or lining the pharynx (the Dorsal, Ventral and Posterior Pharyngeal Sense Organs)^22^,^23^ (Fig. 2A). Neurons in these organs express transcripts and/or genetic reporters for members of three chemosensory families, Odorant Receptors (ORs), Gustatory Receptors (GRs) and Ionotropic Receptors (IRs). ORs appear to be restricted to the Dorsal Organ (where they detect volatile compounds)^24^,^25^, while individual GRs and IRs are expressed in small numbers of neurons in one or more of these chemosensory organs^26^,^27^,^28^,^29^,^30^. Remarkably few relationships have been defined between specific gustatory ligands and particular sensory neurons in the larva^31^,^32^,^33^.

We were particularly interested to test the contribution of IRs to amino acid detection, because these receptors are distantly related to ionotropic glutamate receptors (iGluRs)^27^,^34^,^35^. iGluRs are best known for their role in mediating synaptic transmission, but homologues in prokaryotes and plants have been suggested to function as more broadly-tuned amino acid sensors^36^,^37^. In the adult olfactory and gustatory systems, most IRs are expressed in discrete, functionally specialised neurons^27^,^34^,^38^,^39^, but three (IR8a, IR25a, IR76b) are expressed in multiple neuron classes. These IRs appear to function as co-receptors together with “tuning” IR subunits that define ligand specificity^40^. We reasoned that study of these co-receptors might provide an entry-point to determine the contribution of this repertoire to amino acid sensing.

IR8a expression was not detected in the larva, using available antibodies or genetic reporters^40^ (data not shown). By contrast, consistent with previous analyses^26^,^27^,^28^, we observed expression of IR25a in multiple sensory neurons in all of the larval chemosensory organs (Fig. 2B and data not shown). An IR76b reporter (*IR76b-Gal4*^38^) is expressed in a similarly broad pattern, except the Dorsal Organ, with substantial, but not complete, overlap with the IR25a-positive cells (Fig. 2B and data not shown).

To test the contribution of IR25a and IR76b to amino acid sensing, we examined the responses of mutant larvae lacking these receptor genes to the stimuli that evoked the strongest attraction (aspartic acid, glutamic acid, cysteine and phenylalanine). *IR25a* mutant larvae exhibited statistically indistinguishable responses when compared to control genotypes, indicating that this co-receptor is dispensable for amino acid sensing (Fig. 2C). However, *IR76b* mutants displayed substantially decreased or abolished behavioural responses to aspartic acid, glutamic acid and phenylalanine (Fig. 2D). These defects could be rescued by expression of an *IR76b* cDNA under the control of *IR76b-Gal4*, confirming these neurons as the site of action of this receptor (Fig. 2D). Interestingly, *IR76b* mutants still preferred cysteine (Fig. 2D); we speculate that this response might reflect, in part, a contribution of the olfactory system to detection of this odoriferous amino acid^41^.

### *IR76b-Gal4* neurons respond physiologically to amino acids

**Figure 3:**
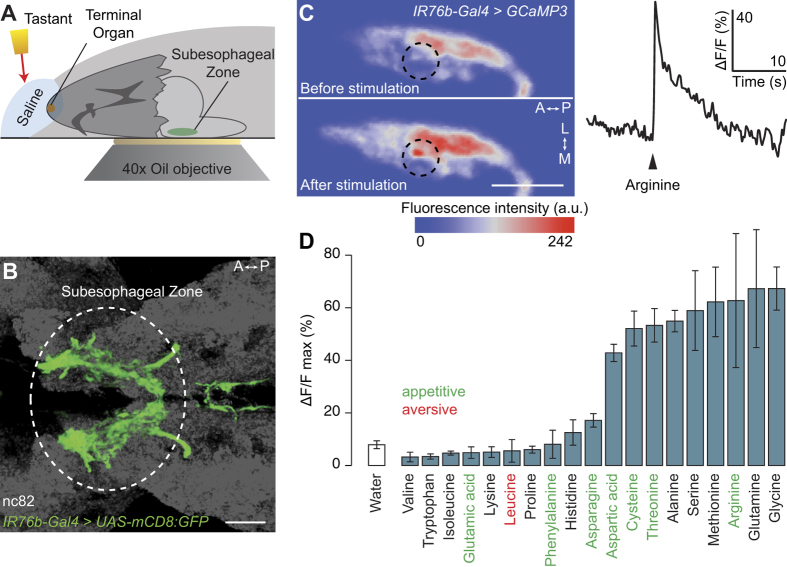
*IR76b-Gal4* neurons respond physiologically to amino acids. **(A)** Schematic of the calcium imaging assay. A larval head is dissected, leaving the brain attached, and embedded in agarose. Calcium responses in the subesophageal zone (SEZ) are measured while a tastant solution is applied to the peripheral chemosensory organs. **(B)** Projections of *IR76b-Gal4* neurons to the SEZ (*IR76b-Gal4;UAS-mCD8:GFP*). The neuropil marker nc82 is shown in grey. Scale bar: 20 μm; A: anterior, P: posterior. **(C)** Left: False-coloured representation of GCaMP3 fluorescence intensity in one hemisphere of the SEZ of a *UAS-GCaMP3;IR76b-Gal4* animal, before and after presentation of 50 mM arginine. Scale bar: 20 μm; L: lateral, M: medial. Right: example time course of the calcium response of *IR76b-Gal4* neurons to arginine presentation (arrowhead) from the region of interest indicated by a dashed circle on the left panel. **(D)** Peak ΔF/F value observed in *IR76b-Gal4* neurons after stimulation with 19 individual amino acids (concentration: 50 mM) or water solvent. (Mean response ± SEM is shown; N ≥ 4 animals per stimulus). The valence of the behavioural responses towards amino acids (from Fig. 1B) is indicated by the colour of lettering.

We next asked whether neurons labelled by *IR76b-Gal4* respond physiologically to amino acids. We developed a preparation that allowed us to measure changes in calcium levels (using the genetically-encoded reporter GCaMP3^42^) in the axonal projections of these neurons in the brain upon application of taste stimuli to the larval head (Fig. 3A). Consistent with the large number of gustatory neurons labelled by *IR76b-Gal4* (Fig. 2B), the corresponding axon termini innervate a broad region of the subesophageal zone (SEZ), their primary target area in the larval brain (Fig. 3B). Application of arginine, an amino acid that elicits preference behaviour in larvae (Fig. 1B), evoked robust increases in calcium levels (Fig. 3C). These calcium changes were spatially restricted to subregions of the axonal innervations, generally within the posterior half of the SEZ, suggesting that only a subset of *IR76b-Gal4* neurons are sensitive to amino acids (Fig. 3C, left). Analysis of the temporal dynamics revealed a fast onset of the response (Fig. 3C, right), although it was not possible to calculate precisely the response latency due to the method of stimulus presentation.

We extended this analysis by measuring responses to 18 other amino acids (tyrosine was excluded because of its low solubility in water). Increases in calcium levels were observed upon presentation of nine amino acids; this subset did not correspond precisely with the stimuli that trigger preference behaviour (Fig. 3D). This discordance might reflect the existence of neurons outside the *IR76b-Gal4* population that contribute to behaviours towards at least some of the amino acids and/or that we are unable to detect physiological responses to certain ligands with our calcium imaging approach.

### Identification of a single neuronal subset responsive to amino acids

**Figure 4:**
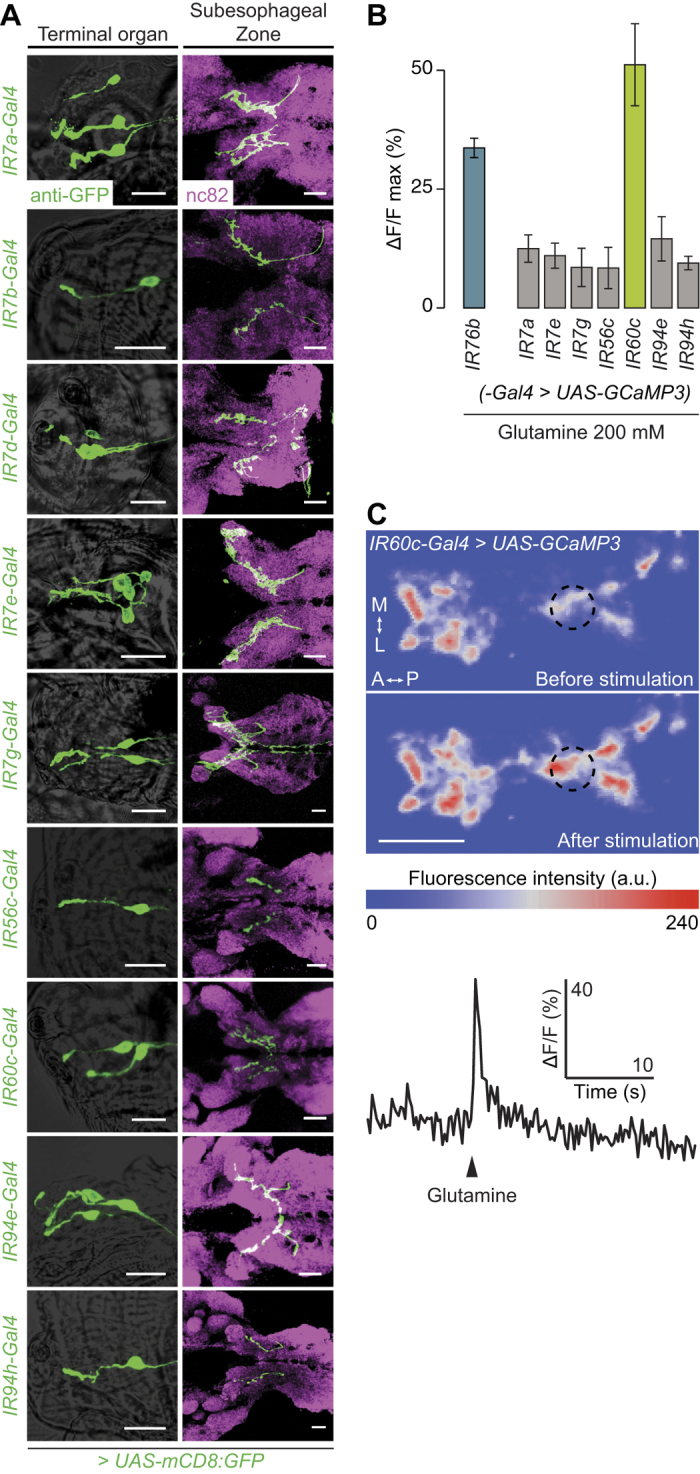
Identification of a single neuronal population responsive to amino acids. **(A)** Expression patterns of nine *IR-Gal4* lines (visualised with a *UAS-mCD8:GFP* reporter) that label subsets of gustatory neurons in the Terminal Organ (genotypes of the form: *UAS-mCD8:GFP;IRxx-Gal4*). Left: soma and dendrites of *IR-Gal4* neurons in the Terminal Organ (anti-GFP, green, overlaid on a brightfield image). Right: projection pattern of *IR-Gal4* neuron axons in the SEZ (anti-GFP, green), overlaid on the neuropil marker nc82 (magenta). Scale bars: 20 μm. **(B)** Peak ΔF/F values of calcium responses measured in the indicated *IR-Gal4* neurons (genotypes of the form: *UAS-GCaMP3;IRxx-Gal4*) to presentation of glutamine (200 mM; preliminary observations prompted our use of higher concentrations than in Fig. 3, to ensure detection of responses in the sparse innervations of individual populations of *IR-Gal4* neurons). *IR7b-Gal4* and *IR7d-Gal4* were excluded because the basal GCaMP3 fluorescence with these drivers was too low to perform imaging experiments. (Mean response ± SEM is shown; N ≥ 5 animals per stimulus). **(C)** Top: False-coloured representation of GCaMP3 fluorescence intensity in one hemisphere of the SEZ of a *UAS-GCaMP3;IR60c-Gal4* animal, before and after presentation of 200 mM glutamine. Scale bar: 20 μm. Bottom: example time course of the calcium response of *IR60c-Gal4* neurons to glutamine presentation (arrowhead) from the region of interest indicated by a dashed circle on the upper panels.

We next sought to characterise in more detail the amino acid-sensitive subset(s) of *IR76b-Gal4* neurons. We reasoned that the fast neuronal responses to amino acids in the imaging experiments would be consistent with a peripheral, rather than internal location of the sensory endings^33^ and therefore focussed our analysis on neurons located in the Terminal Organ. Previous studies identified Gal4 driver lines built from putative regulatory elements of two *IR* genes (*IR7a* and *IR47a*) that are expressed in subsets of Terminal Organ neurons^27^,^28^. In the course of characterisation of a complete set of *IR-Gal4* drivers (V.C. and R.B., unpublished data), we found an additional eight lines (containing regulatory regions for *IR7b, IR7d, IR7e, IR7g, IR56c, IR60c, IR94e* or *IR94h*) that are expressed in 1–4 neurons in the Terminal Organ, and whose axons project to the SEZ (Fig. 4A).

To test whether any of these *IR-Gal4*-expressing neuron populations are sensitive to amino acids, we screened them for calcium responses to glutamine, which evoked one of the strongest responses in *IR76b-Gal4* neurons (Fig. 3D). (We avoided glycine, which produced the highest responses, as this amino acid might also be detected by sweet sensing neurons^43^). For two lines, *IR7b-Gal4* and *IR7d-Gal4*, basal GCaMP3 fluorescence was too weak for reliable measurement of responses. Of those that we were able to analyse, glutamine-evoked calcium increases were detected in only a single population of neurons, labelled by *IR60c-Gal4* (Fig. 4B). These responses displayed a similarly fast onset to those of *IR76b-Gal4* neurons (Fig. 4C).

**Figure 5:**
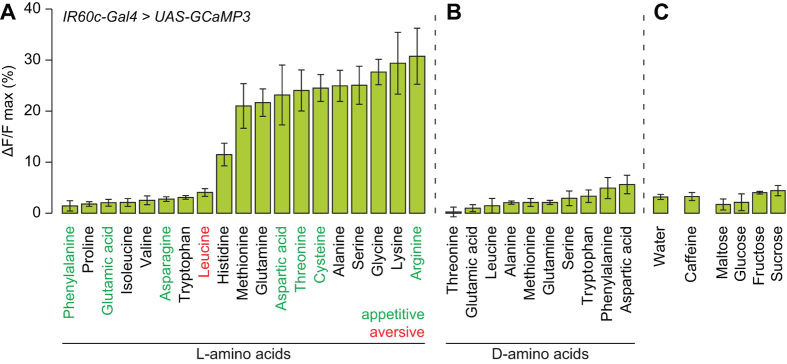
*IR60c-Gal4* neurons are sensitive to a subset of amino acids. **(A)** Peak ΔF/F value observed in neurons of *UAS-GCaMP3;IR60c-Gal4* animals after stimulation with 19 individual L-amino acids (200 mM). (Mean response ± SEM is shown; N ≥ 5 animals per stimulus). **(B)** As in (**A**) after stimulation with 10 individual D-amino acids (200 mM). (Mean response ± SEM is shown; N ≥ 4 animals per stimulus). **(C)** As in (**A**) after stimulation with water, caffeine (50 mM) or sugars (200 mM for maltose, glucose and sucrose; 1 M for fructose). (Mean response ± SEM is shown; N ≥ 3 animals per stimulus).

We expanded our physiological profiling of *IR60c-Gal4* neurons and found that they exhibited strong responses to precisely the same subset of amino acids as *IR76b-Gal4* neurons (Fig. 5A), as well as lysine (potentially reflecting the higher stimulus concentration used in these experiments). Responses were observed for L-isomers but not D-isomers (Fig. 5B), consistent with the neurons only detecting amino acids used in protein synthesis. Moreover, no substantial calcium changes were observed in response to the water solvent or any tested sweet or bitter compound (Fig. 5C).

### *IR60c-Gal4* neurons are not required for amino acid preference

**Figure 6:**
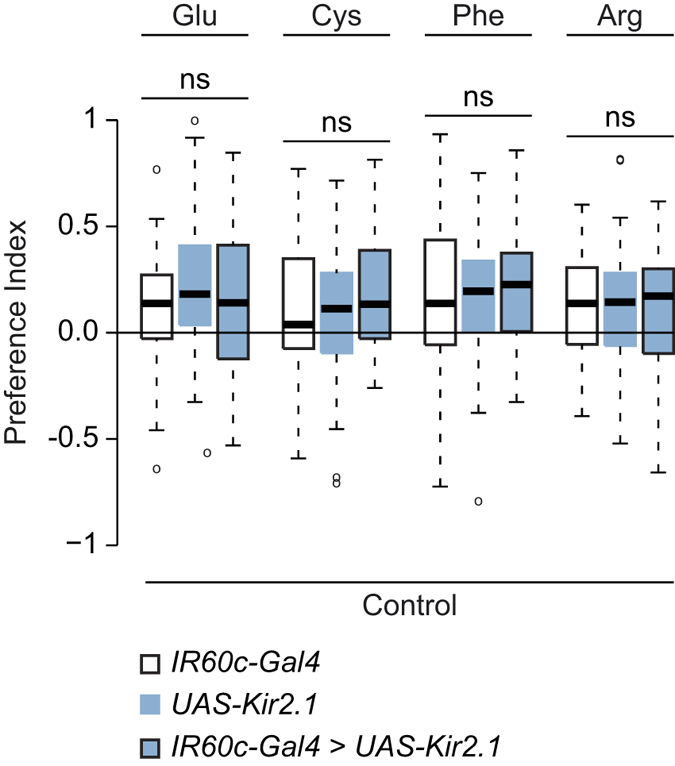
*IR60c-Gal4* neurons are not required for behavioural attraction to amino acids. Preference Indices for four appetitive amino acids (50 mM) of larvae in which *IR60c-Gal4* neurons are silenced with Kir2.1 (*UAS-Kir2.1:GFP/*+*;IR60c-Gal4/*+), together with genetic controls (*UAS-Kir2.1:GFP/*+ and *IR60c-Gal4/*+; “+” represents Canton S-derived chromosomes). (Kruskal-Wallis, p > 0.05; N ≥ 39 groups of ~15 larvae per genotype and per tastant).

As *IR60c-Gal4* neurons were the only identified subpopulation of cells in the Terminal Organ that was activated by amino acids, we tested whether these neurons mediate appetitive behavioural responses of larvae to these compounds. We silenced *IR60c-Gal4* neurons (using the inwardly rectifying potassium channel Kir2.1^44^) and tested the consequence for larval preference for four amino acids that evoke appetitive behaviour (glutamic acid, cysteine, phenylalanine and arginine). Surprisingly, these animals displayed indistinguishable responses compared to genetic controls (Fig. 6), indicating that *IR60c-Gal4* neurons are not required for behavioural attraction to amino acids.

### *IR60c-Gal4* neurons and IR76b mediate feeding suppression by amino acids

**Figure 7:**
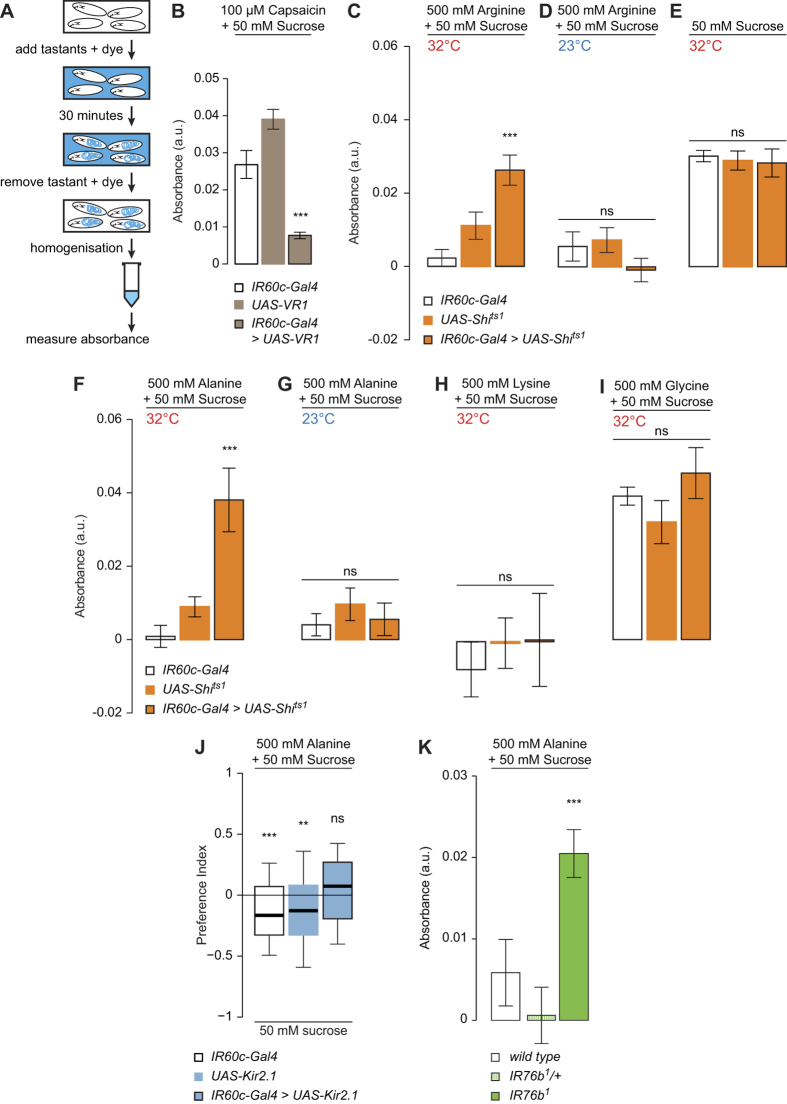
*IR60c-Gal4* neurons and IR76b mediate feeding suppression by amino acids. **(A)** Schematic of the feeding suppression assay. **(B)** Absorbance (at 625 nm) of extracts from homogenised *UAS-VR1/*+*;IR60c-Gal4/*+ larvae and controls (*UAS-VR1/*+ and *IR60c-Gal4/*+; “+” represents Canton S-derived chromosomes) after animals have fed on 50 mM sucrose containing 100 μM capsaicin and 0.4% Brilliant Blue for 30 minutes. (Kruskal-Wallis, ***p < 0.001; N = 11 groups of 30 larvae per genotype). **(C,D)** Absorbance of extracts from homogenised *UAS-Shibire*^*ts1*^*/*+*;;UAS-Shibire*^*ts1*^*/IR60c-Gal4* larvae and controls (*UAS-Shibire*^*ts1*^*/*+*;;UAS-Shibire*^*ts1*^*/*+ and *IR60c-Gal4*/+; “+” represents Canton S-derived chromosomes) after animals have fed on 50 mM sucrose containing 0.4% Brilliant Blue for 30 minutes at the restrictive (**C**) or permissive (**D**) temperature. (Kruskal-Wallis, ***p < 0.001; N = 6 groups of 30 larvae per genotype). **(E)** Absorbance of extracts from homogenised larvae of the same genotypes as in (**C**) after animals have fed on 50 mM sucrose containing 0.4% Brilliant Blue for 30 minutes at the restrictive temperature. (Kruskal-Wallis, p > 0.05; N ≥ 12 groups of 30 larvae per genotype). **(F–G)** Absorbance of extracts from homogenised larvae of the same genotypes as in (**C**) after animals have fed on 50 mM sucrose containing 500 mM alanine and 0.4% Brilliant Blue for 30 minutes at the restrictive (**F**) or permissive (**G**) temperature. (Kruskal-Wallis, ***p < 0.001; N ≥ 12 groups of 30 larvae per genotype). **(H–I)** Absorbance of extracts from homogenised larvae of the same genotypes as in (**C**) after animals have fed on 50 mM sucrose containing 500 mM lysine (**H**) or 500 mM glycine (**I**) and 0.4% Brilliant Blue for 30 minutes at the restrictive temperature. (Kruskal-Wallis, ***p < 0.001; N ≥ 12 groups of 30 larvae per genotype). **(J)** Preference Indices of larvae in which *IR60c-Gal4* neurons are silenced with Kir2.1 (*UAS-Kir2.1:GFP/*+*;IR60c-Gal4/*+), together with controls (*UAS-Kir2.1:GFP/*+ and *IR60c-Gal4/*+; “+” represents Canton S-derived chromosomes) for 50 mM sucrose *versus* 50 mM sucrose +500 mM alanine. (Wilcoxon Signed Rank Test, **p < 0.01, ***p < 0.001; N ≥ 69 groups of ~15 animals per genotype). **(K)** Absorbance of extracts from homogenised *wild-type (w*^*1118*^), homozygous *IR76b*^*1*^ mutant and heterozygous *IR76b*^*1*^*/*+ (“+” represents *w*^*1118*^ derived chromosomes) larvae after animals have fed on 50 mM sucrose containing 500 mM alanine and 0.4% Brilliant Blue for 30 minutes. (Kruskal-Wallis, ***p < 0.001; N = 16 groups of 30 larvae per genotype).

Given the lack of requirement of *IR60c-Gal4* neurons for attraction to amino acids, we surmised that they control other types of behaviour. We therefore developed an experimental paradigm in which larvae are allowed to feed from a sucrose solution containing an edible dye to allow spectrophotometric quantification of food intake (see Methods) (Fig. 7A). The role of *IR60c-Gal4* neurons in modulating such feeding was assessed by artificially activating these cells with VR1, a mammalian ion channel gated by capsaicin (which is a behaviourally neutral compound for *Drosophila*^45^,^46^). We observed substantial feeding on sucrose/capsaicin in genetic controls (Fig. 7B). By contrast, animals expressing VR1 in *IR60c-Gal4* neurons displayed greatly suppressed feeding in the presence of capsaicin (Fig. 7B). This observation suggests that *IR60c-Gal4* neurons mediate an aversive signal that can counteract the appetitiveness of sucrose.

We next tested whether natural amino acid stimuli could also evoke feeding suppression through *IR60c-Gal4* neurons. Indeed, adding a high concentration of arginine to the sucrose solution almost completely inhibited feeding in genetic controls (Fig. 7C,D). This inhibition was lifted when *IR60c-Gal4* neurons were silenced (with Shibire^ts1 ^47) (Fig. 7C), with these animals feeding at similar levels to assays where no amino acid was present (Fig. 7E). This phenotype did not appear to be due to enhanced perception of sucrose, because blocking *IR60c-Gal4* neuron function had no effect on feeding on sucrose alone (Fig. 7E).

We extended this analysis to other amino acid ligands of *IR60c-Gal4* neurons: alanine, lysine and glycine. As observed with arginine, high concentrations of alanine suppressed feeding, and this depended upon functional *IR60c*-*Gal4* neurons (Fig. 7F–G). By contrast, addition of lysine or glycine revealed two different effects. Lysine suppressed feeding even when *IR60c-Gal4* neurons were blocked (Fig. 7H), suggesting that additional sensory pathways mediate feeding suppression by this amino acid. Glycine did not produce any feeding inhibition, even in animals with functional *IR60c-Gal4* neurons (Fig. 7I). Given that glycine can activate *IR60c-Gal4* neurons (Fig. 5A), the latter observation suggests that this amino acid is also sensed by a distinct attraction-mediating pathway that overrides *IR60c*-*Gal4* neuron-dependent aversion.

We confirmed a role for *IR60c-Gal4* neurons in mediating aversion to high concentrations of amino acids in a gustatory choice assay for substrates containing sucrose alone versus sucrose supplemented with a high concentration of alanine (which gave the most robust phenotype in the feeding assay) (Fig. 7F). While control larvae avoided sucrose medium containing this amino acid, animals with silenced *IR60c-Gal4* neurons did not (Fig. 7J).

Finally, we tested whether the putative co-receptor IR76b is also involved in feeding suppression by high concentrations of amino acids. While *wild-type* and heterozygous *IR76b* control strains barely ate sucrose containing amino acids, animals lacking *IR76b* robustly consumed this solution (Fig. 7K). This phenotype is similar to that observed when inhibiting *IR60c-Gal4* neurons. These observations indicate that IR76b is not only required for appetitive behaviour towards amino acids in palatable concentrations (Fig. 2D), but also for feeding suppression by high concentrations of these compounds.

### Distinct pathways for larval aversion

**Figure 8:**
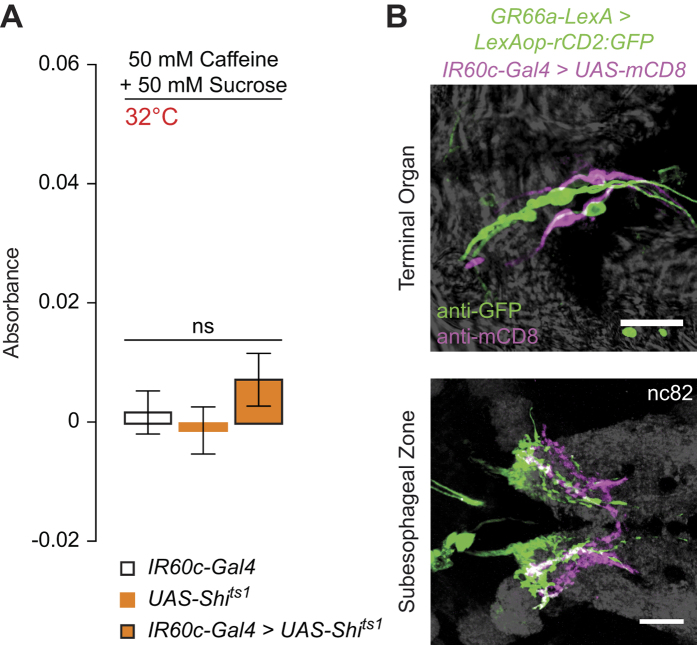
*IR60c-Gal4* neurons define a functionally and anatomically distinct aversion pathway. **(A)** Absorbance (at 625 nm) of extracts from homogenised *UAS-Shibire*^*ts1*^*/*+*;;UAS-Shibire*^*ts1*^*/IR60c-Gal4* larvae and genetic controls (*UAS-Shibire*^*ts1*^*/*+*;;UAS-Shibire*^*ts1*^*/*+ and *IR60c-Gal4*/+; “+” represents Canton S-derived chromosomes) after animals have fed on 50 mM sucrose containing 50 mM caffeine and 0.4% Brilliant Blue FCF for 30 minutes. (Kruskal-Wallis, p > 0.05; N ≥ 12 groups of 30 larvae per genotype). **(B)** Immunofluorescence with anti-GFP (green) and anti-CD8 (magenta) on *GR66a-LexA/*+*;LexAOP-rCD2:GFP/UAS-mCD8;IR60c-Gal4/*+ animals revealing that these aversive pathways define distinct populations of neurons in the Terminal Organ (top) but have partially overlapping axonal innervations in the SEZ (bottom). Grey background shows the brightfield image on the top panel and the neuropil marker nc82 on the bottom panel. Scale bars: 20 μm.

Larval behavioural aversion to bitter compounds, such as caffeine, has been reported upon activation of GR66a-expressing Terminal Organ neurons^45^, prompting us to investigate the role of *IR60c-Gal4* neurons in caffeine-related behaviour. Similar to the effect of high concentrations of amino acids, caffeine potently suppressed larval feeding on sucrose (Fig. 8A). However, this suppression is unaffected by inhibition of *IR60c-Gal4* neurons (Fig. 8A). This result is consistent with our demonstration that caffeine does not activate these neurons (Fig. 5C), and suggests the existence of different pathways for feeding suppression by amino acids and bitter substances. Indeed, anatomical comparison using transgenic reporters for *IR60c* and *GR66a* revealed that there is no overlap between these populations of neurons in the Terminal Organ (Fig. 8B). However, the axonal projections of these neurons partially overlap in the SEZ (Fig. 8B), raising the possibility that they converge on at least some common second-order neurons.

## Discussion

This study is, to our knowledge, the first to investigate the molecular and neuronal mechanisms underlying amino acid detection in the *Drosophila* larva. Despite the numerical simplicity of this animal’s chemosensory system^22^,^23^, our data reveal an unexpected complexity in how it responds to this class of compounds. We find that different amino acids can produce behavioural responses of opposing valence. Moreover an amino acid stimulus that produces appetitive behaviour at low concentration can evoke aversion at higher concentrations. Behavioural valence does not correlate with the essential/non-essential dietary requirements for amino acids, nor with their physico-chemical properties (as observed in an independent study (T. Tanimura, personal communication)). These observations suggest that the assignment of value to amino acids is a complex process, potentially related to the diverse properties and biological functions of these chemicals^1^. It is important to note that the presumed “innate” behaviours we have measured in third instar larvae are not necessarily fixed across life stages and strains of different genetic background (T. Tanimura, personal communication), as previously noted in adult *Drosophila*^48^.

We have identified a single class of broadly (but not universally) tuned amino acid sensing neurons, marked by *IR60c-Gal4*, in the main gustatory organ of the larva. Unexpectedly, the physiological profile of these neurons does not show concordance with the observed behavioural sensitivity or valence for these compounds. This observation implies the existence of additional sensory pathways for amino acids not revealed in the present study, or which exhibit responses below the detection threshold of our experimental assays.

Our demonstration that at least some of the behavioural responses to amino acids require the IR76b co-receptor provides the first evidence for the molecular mechanism of this taste modality. It seems very unlikely, however, that IR76b functions as an amino acid sensor itself. This receptor is broadly expressed in multiple populations of chemosensory neurons, and, in adults, has been implicated in sensory transduction of diverse odours in the olfactory system^40^,^49^, and in polyamine and salt sensing in distinct subsets of neurons in the gustatory system^49^,^50^. IR76b has been suggested to function independently as a salt sensor (potentially by acting as sodium leak channel)^50^, but in other contexts it appears to function as a co-receptor with other more selectively-expressed IRs that determine ligand specificity^40^,^49^. We propose that IR76b must also act with one, or more, different tuning IRs to mediate responses to amino acids. Although we identified the *IR60c-Gal4* expressing neurons as amino acid sensors, IR60c does not itself appear to be required for physiological responses of these cells (V.C., J.R.A. and R.B., unpublished data), suggesting that other receptors act with IR76b in these neurons. Regardless, the implication of a variant iGluR in peripheral amino acid sensing in insects provides an interesting molecular analogy to the role of the mGluR-related T1R1/T1R3 umami sensors in mammals^11^,^12^, and might be reflective of a broader role of the iGluR superfamily in amino acid sensing^36^,^37^.

## Methods

### *Drosophila* strains

All animals were maintained on standard cornmeal-agar medium under a 12 hour light:12 hour dark cycle at 25 °C. *Wild-type* flies were *w*^*1118*^, Oregon R and Canton S, as indicated in the corresponding figure legends. We used the following mutant and transgenic strains: *IR25a*^2 ^34, *IR25a rescue* (BAC CH322-32C20)^51^, *IR76b-Gal4*^38^, *IR76b*^1 ^50, *UAS-IR76b*^50^, *UAS-mCD8:GFP*^52^, *UAS-GCaMP3*^42^, *UAS-Kir2.1:GFP*^44^, *UAS-VR1*^46^, *UAS-Shibire*^*ts1 *^47, *LexAop-rCD2:GFP*^53^, *UAS-mCD8*^53^, *GR66a-LexA*^54^.

### *IR-Gal4* transgenes

Primers were designed to amplify putative regulatory regions (covering the entire 5′ intergenic region) from Oregon R genomic DNA with appropriate flanking restriction sites (Supplementary Table 1). PCR products were cloned in pGEM-T Easy (Promega), end-sequenced, and sub-cloned into the pGal4 *attB* vector^27^. All constructs were integrated into the attP2 landing site at cytological location 68A^55^, by phiC31-mediated transgenesis (Genetic Services, Inc.).

### Behavioural analyses

#### Larval gustatory choice assays

All chemicals were from Sigma-Aldrich or Applichem (Supplementary Table 2). For Figs 1 and 2: a 0.8% agarose solution was prepared and cooled to <50°C before adding the desired quantity of tastant (if any). 12.5 ml of the relevant agarose solution were poured into each of the four compartments of Star™ Dish 90 × 15 mm Petri dishes (VWR): two diametrically-opposed “agarose only” quadrants, and two “agarose + tastant” quadrants. Media were allowed to cool to room temperature and polymerise for >1 hour. Plates were prepared freshly on the same day as behavioural assays. Third instar larvae were collected, rinsed with tap water and kept for ~1 hour on 0.8% agarose. Groups of 30 animals were placed at the centre of each Petri dish under red light. For Figs 6 and 7J: split Petri dishes (60 mm diameter, VWR) were used and the agarose concentration was 1%. Groups of 15 animals were collected and kept for 1 hour in a droplet of tap water. For the assays in Fig. 7J, larvae were used immediately after collection. Animals were placed in the centre of the Petri dish under white light. For all assays, the number of animals on each substrate was counted after 20 minutes and the Preference Index was calculated as shown in Fig. 1A. Statistics and plots were made with R (R-Development-Core-Team; www.R-project.org).

#### Larval feeding assays

Groups of 30 early third instar larvae were collected and kept for 1 hour in 500 μl PBS in 1.5 ml microcentrifuge tubes placed on their sides. Tubes were incubated at the desired permissive (23 °C) or restrictive (32 °C) temperature for an additional 20 minutes. 500 μl of 2X tastant solution and Brilliant Blue FCF (Spectrum Chemical; final concentration 0.4%) in PBS was added to the tubes and larvae were allowed to feed for 30 minutes at the selected temperature. Tubes were transferred to ice for 15 minutes to anesthetise animals and terminate feeding. Larvae were washed 4x with PBS, homogenised in 100 μl PBS and centrifuged for 12 minutes at 13,000 rpm. 50 μl of the supernatant was pipetted into 50 μl PBS and the absorbance of this solution at 625 nm was measured using a NanoDrop. A solution from *wild-type* larvae that had not been fed with dye was used as a blank for the measurement. Plots and analyses were performed with Prism 6 (GraphPad Software; www.graphpad.com).

### Histology

Heads or brains of third instar larvae were dissected in 1XPBS/0.1% Triton (P/T) and immediately fixed in 1XPBS + 4% PFA for >1 hour at 4 °C, then washed 3 × 10 minutes in P/T, blocked for 1 hour in 5% heat-inactivated goat serum in P/T (P/T/S) and incubated 24 hours at 4 °C with primary antibodies diluted in P/T/S. Tissues were washed and blocked again as above, and incubated with secondary antibodies in P/T/S for >24 hours at 4 °C, before final washes as above. Samples were mounted on glass slides in Vectashield (Vector Laboratories, Inc.). Primary antibodies: rabbit anti-IR25a (1:500)^34^, mouse anti-GFP (1:500; Invitrogen), rat anti-mCD8 (1:500; Jackson ImmunoResearch), mouse nc82 (1:10; Developmental Studies Hybridoma Bank). Secondary antibodies (all diluted 1:500): Alexa488 anti-mouse, Cy3 anti-mouse, Alexa488 anti-rabbit, Cy3 anti-rabbit, Cy3 anti-rat, Cy5 anti-rat (Milan Analytica AG). Images were collected with a Zeiss LSM 510 Meta upright confocal microscope (Zeiss, Oberkochen, Germany), using a Plan-APOCHROMAT 63x/1,40 Oil DIC objective. Images were further processed with NIH ImageJ^56^.

### Calcium imaging

A head of a third instar larva expressing *UAS-GCaMP3* under the control of the desired *IR-Gal4* driver was dissected so that the brain remained attached to the mouthparts. Imaginal discs and salivary glands were removed. The head was placed on a 50 μl drop of 1.5% low melting point agarose (Promega) in *Drosophila* sugar-free adult haemolymph-like (AHL) Ringer’s saline (108 mM NaCl, 5 mM KCl, 2 mM CaCl_2_, 8.2 mM MgCl_2_, 4 mM NaHCO_3_, 1 mM NaH_2_PO4, 5 mM HEPES pH 7.5) at 50 °C on a 25 × 60 mm glass coverslip and gently pulled away from the drop so that the brain directly lay on the coverslip. After polymerisation a second 50 μl drop of 1.5% agarose at 50 °C was added on top of sample. Once polymerised, agarose was removed from the tip of the larval head with a sharp blade to uncover peripheral sensory organs, and replaced with 5 μl sugar-free AHL Ringer’s saline, to which an equal volume of tastant solution was added at the appropriate time to stimulate sensory neurons.

The subesophageal zone (SEZ) was visualised with a Zeiss LSM 510 Meta inverted microscope using an EC Plan-NEOFLUAR 40x/1,30 Oil DIC objective. Images were acquired with an AxioCam MRm camera using LSM 3.5 Software. Typically, recordings were made for 87.5 seconds with 4 images per second (350 frames) and tastants were added after 25 seconds (frame 100). As tastants could not be washed from the preparation, only one stimulus was used per animal, except where a tastant did not elicit a response; in these cases, a positive control stimulus (i.e., an established amino acid agonist) was subsequently applied to verify viability and responsiveness of the preparation.

Lateral movement on collected image stacks was corrected using the StackReg plugin (bigwww.epfl.ch/thevenaz/stackreg) in NIH ImageJ. Image stacks were further processed using an adapted custom MATLAB (MathWorks) script^38^. The relative percentage change in fluorescence (ΔF/F) was measured as: (F_i_ − F_0_)/F_0_ × 100, where F_0_ is the mean fluorescence value of frames 20–95 (before stimulus presentation), and F_i_ is the fluorescence value for the *i*^th^ frame. Both sides of the SEZ were imaged and for each sample, the Region of Interest (ROI) was a 10 μm diameter circle around an area displaying high fluorescence changes (determined by visual inspection), which was invariably within the posterior half of one of the two sides of the SEZ projection, where Terminal Organ neuron innervations are located. For each frame, the average ΔF/F was calculated from this ROI. The peak value recorded within the 25 seconds (100 frames) following stimulation was defined as “ΔF/F max”. Plots were produced with custom scripts in R and ImageJ.

## Additional Information

**How to cite this article**: Croset, V. *et al*. A molecular and neuronal basis for amino acid sensing in the *Drosophila* larva. *Sci. Rep.* **6**, 34871; doi: 10.1038/srep34871 (2016).

**Publisher's note:** Springer Nature remains neutral with regard to jurisdictional claims in published maps and institutional affiliations.

## Supplementary Material

Supplementary Information
