# Supplementary material for: A molecular and neuronal basis for amino acid sensing in the Drosophila larva
Source: Sci Rep. 2016 Dec 16;6:34871. doi: 10.1038/srep34871 (PMC5159833; doi:10.1038/srep34871)
Supplement: Supplementary Information [file srep34871-s1.pdf]

A molecular and neuronal basis for amino acid sensing in the *Drosophila* larva

Vincent Croset, Michael Schleyer, J. Roman Arguello, Bertram Gerber and Richard Benton

Supplementary Information

Supplementary Table 1. Construction details of *IR-Gal4* lines.

Supplementary Table 2. Taste stimuli.

Supplementary Table 1. Construction details of *IR-Gal4* lines.

Gene	Forward primer	Reverse primer	Length (bp)	Template	Vector	Integration site
IR7a	AGATCTGGTGAAGAATAGAGTGTGGC	GAATTCTTTGAAACGAAACTGTTGCG	2318	Oregon R genomic DNA	pGal4 *attB*	attP2
IR7b	AGATCTGGGATGAGAACGACATCGAT	GAATTCGGCTAAAGAGTTGCCAAAGG	578	Oregon R genomic DNA	pGal4 *attB*	attP2
IR7d	AGATCTAACTTGTGTCAATGCGATCC	AGATCTGGCGAATGTGAAACATTTGG	1010	Oregon R genomic DNA	pGal4 *attB*	attP2
IR7e	AGATCTTACTTCGGCAGAGGAACTAG	GAATTCTTGCTCCCCGGACAAATCGT	600	Oregon R genomic DNA	pGal4 *attB*	attP2
IR7g	AGATCTATCGATCCTCGAATTCTCCA	AGATCTGTCCGTCTATCGAAATCCGG	766	Oregon R genomic DNA	pGal4 *attB*	attP2
IR56c	AGATCTGCAAAGACGTCCACAGTATG	GAATTCGACTTTCCCTTAGAAGCACC	319	Oregon R genomic DNA	pGal4 *attB*	attP2
IR60c	GAATTCGATTGGATACCACAGGTGGC	GAATTCGGCGACTATCCGAAACGAGC	560	Oregon R genomic DNA	pGal4 *attB*	attP2
IR94e	AGATCTTTGGCGACATAAGATGTGGC	GAATTCTTCCCAGGGGATTACACAAA	322	Oregon R genomic DNA	pGal4 *attB*	attP2
IR94h	GAATTCTTGTTCACGCGGCAATTACG	GAATTCGACTTATACCGAAACCGACG	2000	Oregon R genomic DNA	pGal4 *attB*	attP2

Restriction enzyme sites incorporated into the primers used for PCR amplification are highlighted in blue

Supplementary Table 2. Taste stimuli.

Tastant	CAS	Source	Catalog number	Concentrations
Brilliant Blue FCF	3844-45-9	Spectrum Chemical	FD110	0.40%
Caffeine	58-08-2	Sigma-Aldrich	C0750	50 mM
Capsaicin	404-86-4	Sigma-Aldrich	M2028	100 µM
D-(-)-Fructose	57-48-7	Sigma-Aldrich	F0127	1 M
D-(+)-Glucose	50-99-7	Sigma-Aldrich	G8270	200 mM
D-(+)-Maltose monohydrate	6363-53-7	Sigma-Aldrich	M5885	200 mM
D-alanine	338-69-2	Sigma-Aldrich	A7377	200 mM
D-aspartic acid	1783-96-6	Sigma-Aldrich	219096	200 mM
D-glutamic acid	6893-26-1	Sigma-Aldrich	G1001	200 mM
D-glutamine	5959-95-5	Sigma-Aldrich	G9003	200 mM
D-leucine	328-38-1	Sigma-Aldrich	855448	200 mM
D-methionine	348-67-4	Sigma-Aldrich	M9375	200 mM
D-phenylalanine	673-06-3	Sigma-Aldrich	P1751	200 mM
D-serine	312-84-5	Sigma-Aldrich	S4250	200 mM
D-threonine	632-20-2	Sigma-Aldrich	T8250	200 mM
D-tryptophan	153-94-6	Sigma-Aldrich	T9753	200 mM
D(+)-sucrose	57-50-1	Applichem	A2211	50 mM, 200 mM
Glycine	56-40-6	Sigma-Aldrich	G7126	50 mM, 200 mM, 500 mM
L-alanine	56-41-7	Sigma-Aldrich	A7627	50 mM, 200 mM, 500 mM
L-arginine	74-79-3	Sigma-Aldrich	A5006	50 mM, 200 mM, 500 mM
L-asparagine	70-47-3	Sigma-Aldrich	A0884	50 mM, 200 mM
L-aspartic acid	56-84-8	Sigma-Aldrich	A93100	50 mM, 200 mM
L-cysteine	52-89-1	Sigma-Aldrich	W326305	50 mM, 200 mM
L-glutamic acid	56-86-0	Sigma-Aldrich	G1251	50 mM, 200 mM
L-glutamine	56-85-9	Sigma-Aldrich	G3126	50 mM, 200 mM
L-histidine	71-00-1	Sigma-Aldrich	H8000	50 mM, 200 mM
L-isoleucine	73-32-5	Sigma-Aldrich	W527602	50 mM, 200 mM
L-leucine	61-90-5	Sigma-Aldrich	L8000	50 mM, 200 mM
L-lysine	56-87-1	Sigma-Aldrich	W384704	50 mM, 200 mM, 500 mM
L-methionine	63-68-3	Sigma-Aldrich	M9625	50 mM, 200 mM
L-phenylalanine	63-91-2	Sigma-Aldrich	P2126	50 mM, 200 mM
L-proline	147-85-3	Sigma-Aldrich	W331902	50 mM, 200 mM
L-serine	56-45-1	Sigma-Aldrich	S4500	50 mM, 200 mM
L-threonine	72-19-5	Sigma-Aldrich	T8625	50 mM, 200 mM
L-tryptophan	73-22-3	Sigma-Aldrich	T0254	50 mM, 200 mM
L-tyrosine	60-18-4	Sigma-Aldrich	T3754	2 mM
L-valine	72-18-4	Sigma-Aldrich	V0500	50 mM, 200 mM
